# Supplementary material for: HIVprotI: an integrated web based platform for prediction and design of HIV proteins inhibitors
Source: J Cheminform. 2018 Mar 9;10:12. doi: 10.1186/s13321-018-0266-y (PMC5845081; doi:10.1186/s13321-018-0266-y)
Supplement: Supplementary file 2 — Additional file 2. Source code of HIVProtI web server. [file 13321_2018_266_MOESM2_ESM.zip › HIVprotI_Source-code/hivprotI/weka-3-6-6/doc/weka/gui/Main.html]

Main


---


|  |  |  |
| --- | --- | --- |
| **Overview** \| **Package** \| **Class** \| **Tree** \| **Deprecated** \| **Index** \| **Help** \| **Weka's home** | |  |
| **PREV CLASS**   **NEXT CLASS** | **FRAMES**    **NO FRAMES**     **All Classes** |  |
| SUMMARY: NESTED \| FIELD \| CONSTR \| METHOD | DETAIL: FIELD \| CONSTR \| METHOD |  |


---


## weka.gui Class Main

```
java.lang.Object
  java.awt.Component
      java.awt.Container
          java.awt.Window
              java.awt.Frame
                  javax.swing.JFrame
                      weka.gui.Main
```

**All Implemented Interfaces:**: java.awt.image.ImageObserver, java.awt.MenuContainer, java.io.Serializable, javax.accessibility.Accessible, javax.swing.RootPaneContainer, javax.swing.WindowConstants, OptionHandler

---

``` public class Main extends javax.swing.JFrame implements OptionHandler ```

Menu-based GUI for Weka, replacement for the GUIChooser.
Valid options are:

```
 -gui <MDI|SDI>
  Determines the layout of the GUI:
  MDI = MDI Layout
  SDI = SDI Layout
  (default: MDI)
```

**Version:**
:   $Revision: 7193 $

**Author:**
:   fracpete (fracpete at waikato dot ac dot nz)

**See Also:**: Serialized Form

---

| **Nested Class Summary** | |
| --- | --- |
| `static class` | `Main.BackgroundDesktopPane`             DesktopPane with background image. |
| `static class` | `Main.ChildFrameMDI`             Specialized JInternalFrame class. |
| `static class` | `Main.ChildFrameSDI`             Specialized JFrame class. |

**Nested classes/interfaces inherited from class java.awt.Component**

`java.awt.Component.BaselineResizeBehavior`


| **Field Summary** | |
| --- | --- |
| `static int` | `GUI_MDI`             displays the GUI as MDI. |
| `static int` | `GUI_SDI`             displays the GUI as SDI. |
| `static Tag[]` | `TAGS_GUI`             GUI tags. |

**Fields inherited from class javax.swing.JFrame**

`EXIT_ON_CLOSE`

**Fields inherited from class java.awt.Frame**

`CROSSHAIR_CURSOR, DEFAULT_CURSOR, E_RESIZE_CURSOR, HAND_CURSOR, ICONIFIED, MAXIMIZED_BOTH, MAXIMIZED_HORIZ, MAXIMIZED_VERT, MOVE_CURSOR, N_RESIZE_CURSOR, NE_RESIZE_CURSOR, NORMAL, NW_RESIZE_CURSOR, S_RESIZE_CURSOR, SE_RESIZE_CURSOR, SW_RESIZE_CURSOR, TEXT_CURSOR, W_RESIZE_CURSOR, WAIT_CURSOR`

**Fields inherited from class java.awt.Component**

`BOTTOM_ALIGNMENT, CENTER_ALIGNMENT, LEFT_ALIGNMENT, RIGHT_ALIGNMENT, TOP_ALIGNMENT`

**Fields inherited from interface javax.swing.WindowConstants**

`DISPOSE_ON_CLOSE, DO_NOTHING_ON_CLOSE, HIDE_ON_CLOSE`

**Fields inherited from interface java.awt.image.ImageObserver**

`ABORT, ALLBITS, ERROR, FRAMEBITS, HEIGHT, PROPERTIES, SOMEBITS, WIDTH`


**Constructor Summary**

`Main()`
  
          default constructor.


| **Method Summary** | |
| --- | --- |
| `void` | `addChildFrame(java.awt.Container c)`             adds the given child frame to the list of frames. |
| `static void` | `addStartupListener(StartUpListener s)`             Add a listener to be notified when startup is complete. |
| `boolean` | `containsWindow(java.lang.Class windowClass)`             checks, whether an instance of the given window class is already in the Window list. |
| `boolean` | `containsWindow(java.lang.String title)`             checks, whether a window with the given title is already in the Window list. |
| `static void` | `createSingleton(java.lang.String[] args)`             Create the singleton instance of the Main GUI. |
| `SelectedTag` | `getGUIType()`             Gets the currently set type of GUI to display. |
| `java.lang.String[]` | `getOptions()`             returns the options of the current setup. |
| `static Main` | `getSingleton()`             Return the singleton instance of the Main GUI. |
| `java.awt.Container` | `getWindow(java.lang.Class windowClass)`             returns the first instance of the given window class, null if none can be found. |
| `java.awt.Container` | `getWindow(java.lang.String title)`             returns the first window with the given title, null if none can be found. |
| `java.util.Iterator` | `getWindowList()`             returns all currently open frames. |
| `java.util.Enumeration` | `listOptions()`             Gets an enumeration describing the available options. |
| `static void` | `main(java.lang.String[] args)`             starts the application. |
| `void` | `minimizeWindows()`             minimizes all windows. |
| `boolean` | `removeChildFrame(java.awt.Container c)`             tries to remove the child frame, it returns true if it could do such. |
| `void` | `restoreWindows()`             restores all windows. |
| `void` | `setGUIType(SelectedTag value)`             Sets the type of GUI to use. |
| `void` | `setOptions(java.lang.String[] options)`             Parses the options for this object. |
| `void` | `setVisible(boolean b)`             Shows or hides this component depending on the value of parameter b. |
| `boolean` | `showWindow(java.lang.Class windowClass)`             brings the first frame to the top that is of the specified window class. |
| `boolean` | `showWindow(java.awt.Container c)`             brings child frame to the top. |
| `void` | `windowListChanged()`             is called when window list changed somehow (add or remove). |

**Methods inherited from class javax.swing.JFrame**

`getAccessibleContext, getContentPane, getDefaultCloseOperation, getGlassPane, getGraphics, getJMenuBar, getLayeredPane, getRootPane, getTransferHandler, isDefaultLookAndFeelDecorated, remove, repaint, setContentPane, setDefaultCloseOperation, setDefaultLookAndFeelDecorated, setGlassPane, setIconImage, setJMenuBar, setLayeredPane, setLayout, setTransferHandler, update`

**Methods inherited from class java.awt.Frame**

`addNotify, getCursorType, getExtendedState, getFrames, getIconImage, getMaximizedBounds, getMenuBar, getState, getTitle, isResizable, isUndecorated, remove, removeNotify, setCursor, setExtendedState, setMaximizedBounds, setMenuBar, setResizable, setState, setTitle, setUndecorated`

**Methods inherited from class java.awt.Window**

`addPropertyChangeListener, addPropertyChangeListener, addWindowFocusListener, addWindowListener, addWindowStateListener, applyResourceBundle, applyResourceBundle, createBufferStrategy, createBufferStrategy, dispose, getBufferStrategy, getFocusableWindowState, getFocusCycleRootAncestor, getFocusOwner, getFocusTraversalKeys, getGraphicsConfiguration, getIconImages, getInputContext, getListeners, getLocale, getModalExclusionType, getMostRecentFocusOwner, getOwnedWindows, getOwner, getOwnerlessWindows, getToolkit, getWarningString, getWindowFocusListeners, getWindowListeners, getWindows, getWindowStateListeners, hide, isActive, isAlwaysOnTop, isAlwaysOnTopSupported, isFocusableWindow, isFocusCycleRoot, isFocused, isLocationByPlatform, isShowing, pack, paint, postEvent, removeWindowFocusListener, removeWindowListener, removeWindowStateListener, reshape, setAlwaysOnTop, setBounds, setBounds, setCursor, setFocusableWindowState, setFocusCycleRoot, setIconImages, setLocationByPlatform, setLocationRelativeTo, setMinimumSize, setModalExclusionType, setSize, setSize, show, toBack, toFront`

**Methods inherited from class java.awt.Container**

`add, add, add, add, add, addContainerListener, applyComponentOrientation, areFocusTraversalKeysSet, countComponents, deliverEvent, doLayout, findComponentAt, findComponentAt, getAlignmentX, getAlignmentY, getComponent, getComponentAt, getComponentAt, getComponentCount, getComponents, getComponentZOrder, getContainerListeners, getFocusTraversalPolicy, getInsets, getLayout, getMaximumSize, getMinimumSize, getMousePosition, getPreferredSize, insets, invalidate, isAncestorOf, isFocusCycleRoot, isFocusTraversalPolicyProvider, isFocusTraversalPolicySet, layout, list, list, locate, minimumSize, paintComponents, preferredSize, print, printComponents, remove, removeAll, removeContainerListener, setComponentZOrder, setFocusTraversalKeys, setFocusTraversalPolicy, setFocusTraversalPolicyProvider, setFont, transferFocusBackward, transferFocusDownCycle, validate`

**Methods inherited from class java.awt.Component**

`action, add, addComponentListener, addFocusListener, addHierarchyBoundsListener, addHierarchyListener, addInputMethodListener, addKeyListener, addMouseListener, addMouseMotionListener, addMouseWheelListener, bounds, checkImage, checkImage, contains, contains, createImage, createImage, createVolatileImage, createVolatileImage, disable, dispatchEvent, enable, enable, enableInputMethods, firePropertyChange, firePropertyChange, firePropertyChange, firePropertyChange, firePropertyChange, firePropertyChange, getBackground, getBaseline, getBaselineResizeBehavior, getBounds, getBounds, getColorModel, getComponentListeners, getComponentOrientation, getCursor, getDropTarget, getFocusListeners, getFocusTraversalKeysEnabled, getFont, getFontMetrics, getForeground, getHeight, getHierarchyBoundsListeners, getHierarchyListeners, getIgnoreRepaint, getInputMethodListeners, getInputMethodRequests, getKeyListeners, getLocation, getLocation, getLocationOnScreen, getMouseListeners, getMouseMotionListeners, getMousePosition, getMouseWheelListeners, getName, getParent, getPeer, getPropertyChangeListeners, getPropertyChangeListeners, getSize, getSize, getTreeLock, getWidth, getX, getY, gotFocus, handleEvent, hasFocus, imageUpdate, inside, isBackgroundSet, isCursorSet, isDisplayable, isDoubleBuffered, isEnabled, isFocusable, isFocusOwner, isFocusTraversable, isFontSet, isForegroundSet, isLightweight, isMaximumSizeSet, isMinimumSizeSet, isOpaque, isPreferredSizeSet, isValid, isVisible, keyDown, keyUp, list, list, list, location, lostFocus, mouseDown, mouseDrag, mouseEnter, mouseExit, mouseMove, mouseUp, move, nextFocus, paintAll, prepareImage, prepareImage, printAll, removeComponentListener, removeFocusListener, removeHierarchyBoundsListener, removeHierarchyListener, removeInputMethodListener, removeKeyListener, removeMouseListener, removeMouseMotionListener, removeMouseWheelListener, removePropertyChangeListener, removePropertyChangeListener, repaint, repaint, repaint, requestFocus, requestFocusInWindow, resize, resize, setBackground, setComponentOrientation, setDropTarget, setEnabled, setFocusable, setFocusTraversalKeysEnabled, setForeground, setIgnoreRepaint, setLocale, setLocation, setLocation, setMaximumSize, setName, setPreferredSize, show, size, toString, transferFocus, transferFocusUpCycle`

**Methods inherited from class java.lang.Object**

`equals, getClass, hashCode, notify, notifyAll, wait, wait, wait`

**Methods inherited from interface java.awt.MenuContainer**

`getFont, postEvent`

**Field Detail**

### GUI\_MDI

```
public static final int GUI_MDI
```

:   displays the GUI as MDI.

    **See Also:**: Constant Field Values

---


### GUI\_SDI

```
public static final int GUI_SDI
```

:   displays the GUI as SDI.

    **See Also:**: Constant Field Values

---


### TAGS\_GUI

```
public static final Tag[] TAGS_GUI
```

:   GUI tags.


**Constructor Detail**

### Main

```
public Main()
```

:   default constructor.


**Method Detail**

### addChildFrame

```
public void addChildFrame(java.awt.Container c)
```

:   adds the given child frame to the list of frames.

    :   **Parameters:**: `c` - the child frame to add

---


### removeChildFrame

```
public boolean removeChildFrame(java.awt.Container c)
```

:   tries to remove the child frame, it returns true if it could do such.

    :   **Parameters:**: `c` - the child frame to remove **Returns:**: true if the child frame could be removed

---


### showWindow

```
public boolean showWindow(java.awt.Container c)
```

:   brings child frame to the top.

    :   **Parameters:**: `c` - the frame to activate **Returns:**: true if frame was activated

---


### showWindow

```
public boolean showWindow(java.lang.Class windowClass)
```

:   brings the first frame to the top that is of the specified
    window class.

    :   **Parameters:**: `windowClass` - the class to display the first child for **Returns:**: true, if a child was found and brought to front

---


### getWindowList

```
public java.util.Iterator getWindowList()
```

:   returns all currently open frames.

    :   **Returns:**: an iterator over all currently open frame

---


### getWindow

```
public java.awt.Container getWindow(java.lang.Class windowClass)
```

:   returns the first instance of the given window class, null if none can be
    found.

    :   **Parameters:**: `windowClass` - the class to retrieve the first instance for **Returns:**: null, if no instance can be found

---


### getWindow

```
public java.awt.Container getWindow(java.lang.String title)
```

:   returns the first window with the given title, null if none can be
    found.

    :   **Parameters:**: `title` - the title to look for **Returns:**: null, if no instance can be found

---


### containsWindow

```
public boolean containsWindow(java.lang.Class windowClass)
```

:   checks, whether an instance of the given window class is already in
    the Window list.

    :   **Parameters:**: `windowClass` - the class to check for an instance in the current window list **Returns:**: true if the class is already listed in the Window list

---


### containsWindow

```
public boolean containsWindow(java.lang.String title)
```

:   checks, whether a window with the given title is already in
    the Window list.

    :   **Parameters:**: `title` - the title to check for in the current window list **Returns:**: true if a window with the given title is already listed in the Window list

---


### minimizeWindows

```
public void minimizeWindows()
```

:   minimizes all windows.

---


### restoreWindows

```
public void restoreWindows()
```

:   restores all windows.

---


### windowListChanged

```
public void windowListChanged()
```

:   is called when window list changed somehow (add or remove).

---


### setVisible

```
public void setVisible(boolean b)
```

:   Shows or hides this component depending on the value of parameter b.

    :   **Overrides:**: `setVisible` in class `java.awt.Window`
    :   **Parameters:**: `b` - if true, shows this component; otherwise, hides this component

---


### createSingleton

```
public static void createSingleton(java.lang.String[] args)
```

:   Create the singleton instance of the Main GUI.

    :   **Parameters:**: `args` - commandline options

---


### getSingleton

```
public static Main getSingleton()
```

:   Return the singleton instance of the Main GUI.

    :   **Returns:**: the singleton instance

---


### addStartupListener

```
public static void addStartupListener(StartUpListener s)
```

:   Add a listener to be notified when startup is complete.

    :   **Parameters:**: `s` - a listener to add

---


### listOptions

```
public java.util.Enumeration listOptions()
```

:   Gets an enumeration describing the available options.

    :   **Specified by:**: `listOptions` in interface `OptionHandler`
    :   **Returns:**: an enumeration of all the available options.

---


### getOptions

```
public java.lang.String[] getOptions()
```

:   returns the options of the current setup.

    :   **Specified by:**: `getOptions` in interface `OptionHandler`
    :   **Returns:**: the current options

---


### setOptions

```
public void setOptions(java.lang.String[] options)
                throws java.lang.Exception
```

:   Parses the options for this object.


    Valid options are:

    ```
     -gui <MDI|SDI>
      Determines the layout of the GUI:
      MDI = MDI Layout
      SDI = SDI Layout
      (default: MDI)
    ```

    :   **Specified by:**: `setOptions` in interface `OptionHandler`
    :   **Parameters:**: `options` - the options to use **Throws:**: `java.lang.Exception` - if setting of options fails

---


### setGUIType

```
public void setGUIType(SelectedTag value)
```

:   Sets the type of GUI to use.

    :   **Parameters:**: `value` - .the GUI type

---


### getGUIType

```
public SelectedTag getGUIType()
```

:   Gets the currently set type of GUI to display.

    :   **Returns:**: the current GUI Type.

---


### main

```
public static void main(java.lang.String[] args)
```

:   starts the application.

    :   **Parameters:**: `args` - the commandline arguments - ignored


---


|  |  |  |
| --- | --- | --- |
| **Overview** \| **Package** \| **Class** \| **Tree** \| **Deprecated** \| **Index** \| **Help** \| **Weka's home** | |  |
| **PREV CLASS**   **NEXT CLASS** | **FRAMES**    **NO FRAMES**     **All Classes** |  |
| SUMMARY: NESTED \| FIELD \| CONSTR \| METHOD | DETAIL: FIELD \| CONSTR \| METHOD |  |


---
